# Conditional Rényi Entropy and the Relationships between Rényi Capacities

**DOI:** 10.3390/e22050526

**Published:** 2020-05-06

**Authors:** Gautam Aishwarya, Mokshay Madiman

**Affiliations:** Department of Mathematical Sciences, University of Delaware, Newark, DE 19716, USA; madiman@udel.edu

**Keywords:** conditional Rényi entropy, Rényi mutual information, Rényi capacity, Sibson mutual information

## Abstract

The analogues of Arimoto’s definition of conditional Rényi entropy and Rényi mutual information are explored for abstract alphabets. These quantities, although dependent on the reference measure, have some useful properties similar to those known in the discrete setting. In addition to laying out some such basic properties and the relations to Rényi divergences, the relationships between the families of mutual informations defined by Sibson, Augustin-Csiszár, and Lapidoth-Pfister, as well as the corresponding capacities, are explored.

## 1. Introduction

Shannon’s information measures (entropy, conditional entropy, the Kullback divergence or relative entropy, and mutual information) are ubiquitous both because they arise as operational fundamental limits of various communication or statistical inference problems, and because they are functionals that have become fundamental in the development and advancement of probability theory itself. Over a half century ago, Rényi [1] introduced a family of information measures extending those of Shannon [2], parametrized by an order parameter α∈[0,∞]. Rényi’s information measures are also fundamental– indeed, they are (for α>1) just monotone functions of $L^{p}$-norms, whose relevance or importance in any field that relies on analysis need not be justified. Furthermore, they show up in probability theory, PDE, functional analysis, additive combinatorics, and convex geometry (see, e.g., [3,4,5,6,7,8,9]), in ways where understanding them as information measures instead of simply as monotone functions of $L^{p}$-norms is fruitful. For example, there is an intricate story of parallels between entropy power inequalities (see, e.g., [10,11,12]), Brunn-Minkowski-type volume inequalities (see, e.g., [13,14,15]) and sumset cardinality (see, e.g., [16,17,18,19,20]), which is clarified by considering logarithms of volumes and Shannon entropies as members of the larger class of Rényi entropies. It is also recognized now that Rényi’s information measures show up as fundamental operational limits in a range of information-theoretic or statistical problems (see, e.g., [21,22,23,24,25,26,27]). Therefore, there has been considerable interest in developing the theory surrounding Rényi’s information measures (which is far less well developed than the Shannon case), and there has been a steady stream of recent papers [27,28,29,30,31,32,33,34,35,36] elucidating their properties beyond the early work of [37,38,39,40]. This paper, part of which was presented at ISIT 2019 [41], is a further contribution along these lines.

More specifically, three notions of Rényi mutual information have been considered in the literature (usually named after Sibson, Arimoto and Csiszár) for discrete alphabets. Sibson’s definition has also been considered for abstract alphabets, but Arimoto’s definition has not. Indeed Verdú [31] asserts: “One shortcoming of Arimoto’s proposal is that its generalization to non-discrete alphabets is not self-evident.” The reason it is not self-evident is because although there is an obvious generalized definition, the mutual information arising from this notion depends on the choice of reference measure on the abstract alphabet, which is not a desirable property. Nonetheless, the perspective taken in this note is that it is still interesting to develop the properties of the abstract Arimoto conditional Rényi entropy keeping in mind the dependence on reference measure. The Sibson definition is then just a special case of the Arimoto definition where we choose a particular, special reference measure.

Our main motivation comes from considering various notions of Rényi capacity. While certain equivalences have been shown between various such notions by Csiszár [21] for finite alphabets and Nakiboğlu [36,42] for abstract alphabets, the equivalences and relationships are further extended in this note.

This paper is organized in the following manner. In Section 2 below we begin by defining conditional Rényi entropy for random variables taking values in a Polish space. Section 3 presents a variational formula for the conditional Rényi entropy in terms of Rényi divergence, which will be a key ingredient in several results later. Basic properties that the abstract conditional Rényi entropy satisfies akin to its discrete version are proved in Section 4, including description for special orders 0,1 and *∞*, monotonicity in the order, reduction of entropy upon conditioning, and a version of the chain rule. Section 5 discusses and compares several notions of α-mutual information. The various notions of channel capacity arising out of different notions of α-mutual information are studied in Section 6, which are then compared using results from the preceding section.

## 2. Definition of Conditional Rényi Entropies

Let *S* be a Polish space and $B_{S}$ its Borel σ-algebra. We fix a σ-finite reference measure γ on $\left(S,B_{S}\right)$. Our study of entropy and in particular all $L^{p}$ spaces we talk about will be with respect to this measure space, unless stated otherwise.

**Definition** **1.** *Let X be an S-valued random variable with density f with respect to γ. We define the Rényi entropy of X of order* α∈(0,1)∪(1,∞) *by*

$$h_{\alpha }^{\gamma }\left(X\right)=\frac{\alpha }{1-\alpha }\log{∥f∥}_{\alpha }.$$

It will be convenient to write down Rényi entropy as

$$h_{\alpha }^{\gamma }\left(X\right)=-\log{\mathrm{Ren}}_{\alpha }^{\gamma }\left(X\right),$$

where ${\mathrm{Ren}}_{\alpha }^{\gamma }\left(X\right)={∥f∥}_{\alpha }^{\frac{\alpha }{\alpha -1}}$ will be called the Rényi probability of order α of *X*.

Let *T* be another Polish space with a fixed measure η on its Borel σ-algebra $B_{T}$. Now suppose X,Y are, resepectively, S,T-valued random variables with a joint density F:S×T→R w.r.t. the reference γ⊗η. We will denote the marginals of *F* on *S* and *T* by *f* and *g* respectively. This in particular means that *X* has density *f* w.r.t. γ and *Y* has density *g* w.r.t. η. Just as Rényi probability of *X*, one can define the Rényi probability of the conditional *X* given y=y by the expression ${\mathrm{Ren}}_{\alpha }^{\gamma }\left(X|Y=y\right)={∥\frac{F(\cdot ,y)}{g(y)}∥}_{\alpha }^{\frac{\alpha }{\alpha -1}}$. The following generalizes ([30], Definition 2).

**Definition** **2.** *Let*α∈(0,1)∪(1,∞). *We define the conditional Rényi entropy *$h_{\alpha }^{\gamma }\left(X|Y\right)$*in terms of a weighted mean of conditional Rényi probabilities*${\mathrm{Ren}}_{\alpha }^{\gamma }\left(X|Y=y\right)$,

$$h_{\alpha }^{\gamma }\left(X|Y\right)=-\log{\mathrm{Ren}}_{\alpha }^{\gamma }\left(X|Y\right),$$

*where*

$$\begin{array}{c} {\mathrm{Ren}}_{\alpha }^{\gamma }\left(X|Y\right)={\left(\int_{T}{\mathrm{Ren}}_{\alpha }^{\gamma }{\left(X|Y=y\right)}^{\frac{\alpha -1}{\alpha }}dP_{Y}\right)}^{\frac{\alpha }{\alpha -1}} \end{array}$$

We can re-write ${\mathrm{Ren}}_{\alpha }^{\gamma }\left(X|Y\right)$ as

$${\mathrm{Ren}}_{\alpha }^{\gamma }\left(X|Y\right)={\left(\int_{\mathrm{supp}(P_{Y})}g\left(y\right){\left(\int_{S}{\left(\frac{F(x,y)}{g(y)}\right)}^{\alpha }d\gamma \left(x\right)\right)}^{\frac{1}{\alpha }}d\eta \left(y\right)\right)}^{\frac{\alpha }{\alpha -1}},$$

which is the expected $L^{\alpha }\left(S,\gamma \right)$ norm of the conditional density under the measure $P_{Y}$ raised to a power which is the Hölder conjugate of α. Using Fubini’s theorem the formula for ${\mathrm{Ren}}_{\alpha }^{\gamma }\left(X|Y\right)$ can be further written down only in terms of the joint density,

$${\mathrm{Ren}}_{\alpha }^{\gamma }\left(X|Y\right)={\left(\int_{T}{\left(\int_{S}F{\left(x,y\right)}^{\alpha }d\gamma \left(x\right)\right)}^{\frac{1}{\alpha }}d\eta \left(y\right)\right)}^{\frac{\alpha }{\alpha -1}}.$$

**Remark** **1.** *Suppose*$P_{X|Y=y}$, *for each *y∈supp(g), *denotes the conditional distribution of X given*Y=y, *i.e., the probability measure on S with density *F(x,y)/g(y)*with respect to γ, then the conditional Rényi entropy can be written as*

$$h_{\alpha }^{\gamma }\left(X|Y\right)=\frac{\alpha }{1-\alpha }\log\int_{T}e^{-\frac{1-\alpha }{\alpha }D_{\alpha }\left(P_{X|Y=y}∥\gamma \right)}dP_{Y}\left(y\right),$$

*where $D_{\alpha }\left(\cdot ∥\cdot \right)$ denotes Rényi divergence (see Definition 3).*

When *X* and *Y* are independent random variables one can easily check that ${\mathrm{Ren}}_{\alpha }^{\gamma }\left(X|Y\right)={\mathrm{Ren}}_{\alpha }^{\gamma }\left(X\right)$, therefore $h_{\alpha }^{\gamma }\left(X|Y\right)=h_{\alpha }^{\gamma }\left(X\right)$ as expected. Since the independence of *X* and *Y* means that all the conditionals $P_{X|Y=y}$ are equal to $P_{X}$, the fact that $h_{\alpha }^{\gamma }\left(X|Y\right)=h_{\alpha }^{\gamma }\left(X\right)$ in this case can also be verified from the expression in Remark 1. The converse is also true, i.e., $h_{\alpha }^{\gamma }\left(X|Y\right)=h_{\alpha }^{\gamma }\left(X\right)$ implies the independence of *X* and *Y*, if α≠0,∞. This is noted later in Corollary 2.

Clearly, unlike conditional Shannon entropy, the conditional Rényi entropy is not the average Rényi entropy of the conditional distribution. The average Rényi entropy of the conditional distribution,

$${\tilde{h}}_{\alpha }^{\gamma }\left(X|Y\right):=E_{Y}h_{\alpha }^{\gamma }\left(X|Y=y\right),$$

has been proposed as a candidate for conditional Rényi entropy, however it does not satisfy some properties one would expect such a notion to satisfy, like monotonicity (see [30]). When α≥1 it follows from Jensen’s inequality that $h_{\alpha }^{\gamma }\left(X|Y\right)\leq {\tilde{h}}_{\alpha }^{\gamma }\left(X|Y\right)$, while the inequality is reversed when 0<α<1.

## 3. Relation to Rényi Divergence

We continue to consider an *S*-valued random variable *X* and a *T*-valued random variable *Y* with a given joint distribution $P_{X,Y}$ with density *F* with respect to γ⊗η. Densities, etc are with respect to the fixed reference measures on the state spaces, unless mentioned otherwise.

Let μ be a Borel probability measure with density *p* on a Polish space Ω and let ν be a Borel measure with density *q* on the same space with respect to a common measure γ.

**Definition** **3** (Rényi divergence). *Suppose α∈(0,1)∪(1,∞). Then, the Rényi divergence of order α between measures μ and ν is defined as*

$$D_{\alpha }\left(\mu ∥\nu \right)=\log{\left(\int_{\Omega }p{\left(x\right)}^{\alpha }q{\left(x\right)}^{1-\alpha }d\gamma \left(x\right)\right)}^{\frac{1}{\alpha -1}}.$$

For order 0,1,∞, the Rényi divergence is defined by the respective limits.

**Definition** **4.**

*1*. $D_{0}\left(\mu ∥\nu \right):=\lim_{\alpha \to 0}D_{\alpha }\left(\mu ∥\nu \right)=-\log\nu \left(\mathrm{supp}\left(p\right)\right)$;
*2*. $D_{1}\left(\mu ∥\nu \right):=D\left(\mu ∥\nu \right)=\int p\left(x\right)\log\frac{p(x)}{q(x)}d\gamma \left(x\right)$; *and*
*3*. $D_{\infty }\left(\mu ∥\nu \right):=\lim_{\alpha \to \infty }D_{\alpha }\left(\mu ∥\nu \right)=\log\left(\mathrm{ess}\sup_{\mu }\frac{p(x)}{q(x)}\right).$

**Remark** **2.** *These definitions are independent of the reference measure γ.*

**Remark** **3.** $\lim_{\alpha \to 1}D_{\alpha }\left(\mu ∥\nu \right)=1$*if some *$D_{\alpha }\left(\mu ∥\nu \right)<\infty$. *See [29].*

The conditional Rényi entropy can be written in terms of Rényi divergence from the joint distribution using a generalized Sibson’s identity we learnt from B. Nakiboğlu [43] (also see [36], and [38] where this identity for α≠1 appears to originate from). The proof for abstract alphabets presented here is also due to B. Nakiboğlu [43], which simplifies our original proof [41] of the second formula below.

**Theorem** **1.** *Let*X,Y*be random variables taking vaules in spaces*S,T*respectively. We assume they are jointly distributed with density F with respect to the product reference measure*γ⊗η. *For *α∈(0,∞), *and any probability measure λ absolutely continuous with respect to η, we have*

$$D_{\alpha }\left(P_{X,Y}∥\gamma ⊗\lambda \right)=D_{\alpha }\left(P_{X,Y}∥\gamma ⊗q_{⋆}\right)+D_{\alpha }\left(q_{⋆}∥\lambda \right)=-h_{\alpha }^{\gamma }\left(X|Y\right)+D_{\alpha }\left(q_{⋆}∥\lambda \right),$$

*where*$q_{⋆}=\frac{\mu_{⋆}}{∥\mu_{⋆}∥}$, $\mu_{⋆}$*is the measure having density*$\phi \left(y\right)={\left(\int_{S}F{\left(x,y\right)}^{\alpha }d\gamma \left(x\right)\right)}^{\frac{1}{\alpha }}$*with respect to η, and*$∥\mu_{⋆}∥=\int_{T}{\left(\int_{S}F{\left(x,y\right)}^{\alpha }d\gamma \left(x\right)\right)}^{\frac{1}{\alpha }}d\eta \left(y\right)$*is the normalization factor.*

*As a consequence, we have*

$$h_{\alpha }^{\gamma }\left(X|Y\right)=-\min_{\lambda \in P(T)}D_{\alpha }\left(P_{X,Y}∥\gamma ⊗\lambda \right).$$

**Proof.**  Suppose λ has density *h* with respect to η. Then γ⊗λ has density h(y) with respect to dγ(x)dη(y). Now, for α≠1,

$$\begin{array}{ll} D_{\alpha }\left(P_{X,Y}∥\gamma ⊗\lambda \right) & =\frac{1}{\alpha -1}\log\int_{T}\int_{S}F{\left(x,y\right)}^{\alpha }h{\left(y\right)}^{1-\alpha }d\gamma \left(x\right)d\eta \left(y\right)\\ & =\frac{1}{\alpha -1}\log\int_{T}h{\left(y\right)}^{1-\alpha }\int_{S}F{\left(x,y\right)}^{\alpha }d\gamma \left(x\right)d\eta \left(y\right)\\ & =\frac{1}{\alpha -1}\log\int_{T}h{\left(y\right)}^{1-\alpha }\phi {\left(y\right)}^{\alpha }d\eta \left(y\right)\\ & =\frac{1}{\alpha -1}\log\int_{T}{\left(\frac{d\lambda }{d\eta }\right)}^{1-\alpha }{\left(\frac{d\mu_{⋆}}{d\eta }\right)}^{\alpha }d\eta \left(y\right)\\ & =\frac{1}{\alpha -1}\log\int_{T}{\left(\frac{d\lambda }{d\eta }\right)}^{1-\alpha }{\left(\frac{dq_{⋆}}{d\eta }\right)}^{\alpha }{∥\mu_{⋆}∥}^{\alpha }d\eta \left(y\right)\\ & =\frac{\alpha }{\alpha -1}\log∥\mu_{⋆}∥+\frac{1}{\alpha -1}\log\int_{T}{\left(\frac{d\lambda }{d\eta }\right)}^{1-\alpha }{\left(\frac{dq_{⋆}}{d\eta }\right)}^{\alpha }d\eta \left(y\right)\\ & =\frac{\alpha }{\alpha -1}\log∥\mu_{⋆}∥+D_{\alpha }\left(q_{⋆}∥\lambda \right)\\ & =-h_{\alpha }^{\gamma }\left(X|Y\right)+D_{\alpha }\left(q_{⋆}∥\lambda \right). \end{array}$$

The case α=1 is straightforward and well-known, and the optimal $q_{⋆}$ in this case is the distribution of *Y*.□

**Remark** **4.** *The identities above and the measure* $q_{⋆}$ *are independent of the reference measure η. η is only used to write out the Rényi divergence concretely in terms of densities.*

## 4. Basic Properties

### 4.1. Special Orders

We will now look at some basic properties of the conditional Rényi entropy we have defined above. First we see that the conditional Rényi entropy is consistent with the notion of conditional Shannon entropy of *X* given *Y* defined by

$$h^{\gamma }\left(X|Y\right)=-\int_{T}\int_{S}F\left(x,y\right)\log\frac{F(x,y)}{g(y)}d\gamma \left(x\right)d\eta \left(y\right).$$

**Proposition** **1.** $$\lim_{\alpha \to 1^{+}}h_{\alpha }^{\gamma }\left(X|Y\right)=h_{1}^{\gamma }\left(X|Y\right)=h^{\gamma }\left(X|Y\right),$$

*if*$h_{\alpha }^{\gamma }\left(X|Y\right)<\infty$*for some*α>1.

**Proof.**  We will use the formula in Theorem 1. By the monotonicity in the order α of $h_{\alpha }\left(X|Y\right)$, all limits $\lim_{\alpha \to 1^{+}},\lim_{\alpha \to 1^{-}},\lim_{\alpha \to 0}=\lim_{\alpha \to 0^{+}}$ exist. Furthermore, for every η,

$$\min_{\lambda }D_{\alpha }\left(P_{X,Y}∥\gamma ⊗\lambda \right)\leq D_{\alpha }\left(P_{X,Y}∥\gamma ⊗\eta \right),$$

so

$$\lim_{\alpha \to 1^{+}}\min_{\lambda }D_{\alpha }\left(P_{X,Y}∥\gamma ⊗\lambda \right)\leq \lim_{\alpha \to 1^{+}}D_{\alpha }\left(P_{X,Y}∥\gamma ⊗\eta \right),$$

that is,

$$-\lim_{\alpha \to 1^{+}}h_{\alpha }^{\gamma }\left(X|Y\right)\leq D\left(P_{X,Y}∥\gamma ⊗\eta \right).$$

Now by minimizing over η and hitting both sides with a minus sign yields,

$$\lim_{\alpha \to 1^{+}}h_{\alpha }^{\gamma }\left(X|Y\right)\geq h^{\gamma }\left(X|Y\right).$$

Suppose α≥1, then by nondecreasing-ness of the Rényi divergence in order, for every λ we have

$$D_{\alpha }\left(P_{X,Y}∥\gamma ⊗\lambda \right)\geq D\left(P_{X,Y}∥\gamma ⊗\lambda \right),$$

and so by minimization over λ and hitting with minus signs we obtain $h_{\alpha }^{\gamma }\left(X|Y\right)\leq h^{\gamma }\left(X|Y\right)$. This shows that

$$\lim_{\alpha \to 1^{+}}h_{\alpha }^{\gamma }\left(X|Y\right)=h^{\gamma }\left(X|Y\right).$$

□

We can extend our definition of Rényi probability of order α to α=0 by taking limits, thereby obtaining

$${\mathrm{Ren}}_{0}^{\gamma }\left(X\right)=\frac{1}{\gamma (\mathrm{supp}(f))}.$$

In the next proposition we define the conditional Rényi entropy of order 0 and record a consequence.

**Proposition** **2.** $$h_{0}^{\gamma }\left(X|Y\right):={∥h_{0}^{\gamma }\left(X|Y=y\right)∥}_{L^{\infty }\left(P_{Y}\right)}=\lim_{\alpha \to 0}h_{\alpha }^{\gamma }\left(X|Y\right)$$

**Proof.**  We will again use the formula from 1 in this proof. Just as in the last proof, for every probability measure η,

$$\min_{\lambda }D_{\alpha }\left(P_{X,Y}∥\gamma ⊗\lambda \right)\leq D_{\alpha }\left(P_{X,Y}∥\gamma ⊗\eta \right),$$

so

$$\lim_{\alpha \to 0}\min_{\lambda }D_{\alpha }\left(P_{X,Y}∥\gamma ⊗\lambda \right)\leq \lim_{\alpha \to 0}D_{\alpha }\left(P_{X,Y}∥\gamma ⊗\eta \right),$$

that is,

$$-\lim_{\alpha \to 0}h_{\alpha }^{\gamma }\left(X|Y\right)\leq D_{0}\left(P_{X,Y}∥\gamma ⊗\eta \right).$$

Now by minimizing over η and hitting both sides with a minus sign yields,

$$\lim_{\alpha \to 0}h_{\alpha }^{\gamma }\left(X|Y\right)\geq h_{0}^{\gamma }\left(X|Y\right).$$

Suppose α≥0, then by nondecreasing-ness of the Rényi divergence in order, for every λ we have

$$D_{\alpha }\left(P_{X,Y}∥\gamma ⊗\lambda \right)\geq D_{0}\left(P_{X,Y}∥\gamma ⊗\lambda \right),$$

and so by minimization over λ and hitting with minus signs we obtain $h_{\alpha }^{\gamma }\left(X|Y\right)\leq h_{0}^{\gamma }\left(X|Y\right)$. This shows that

$$\lim_{\alpha \to 0}h_{\alpha }^{\gamma }\left(X|Y\right)=h_{0}^{\gamma }\left(X|Y\right).$$

□

### 4.2. Monotonicity in Order

The (unconditional) Rényi entropy decreases with order, and the same is true of the conditional version.

**Proposition** **3.** *For random variables X and Y,*

$$h_{\beta }^{\gamma }\left(X|Y\right)\leq h_{\alpha }^{\gamma }\left(X|Y\right),$$

*for all*0<α≤β≤∞.

The proof is essentially the same as in the discrete setting, and follows from Jensen’s inequality.

**Proof.**  We obtain separately the cases 1<α≤β<∞, 0<α≤β<1, so that the entire Proposition follows from taking limits and the transitivity of inequality. Let α≤β be positive numbers, set $e=\frac{\beta -1}{\beta }\frac{\alpha }{\alpha -1}$. Consider the following argument.

$$\begin{array}{cc} {\mathrm{Ren}}_{\beta }\left(X|Y\right) & ={\left(\int_{\mathrm{supp}(P_{Y})}{\mathrm{Ren}}_{\beta }^{\gamma }{\left(X|Y=y\right)}^{\frac{\beta -1}{\beta }}dP_{Y}\right)}^{\frac{\beta }{\beta -1}}\\ & \geq {\left(\int_{\mathrm{supp}(P_{Y})}{\mathrm{Ren}}_{\alpha }^{\gamma }{\left(X|Y=y\right)}^{\frac{\beta -1}{\beta }}dP_{Y}\right)}^{\frac{\beta }{\beta -1}}\\ & ={\left(\int_{\mathrm{supp}(P_{Y})}{\mathrm{Ren}}_{\alpha }^{\gamma }{\left(X|Y=y\right)}^{\frac{\alpha -1}{\alpha }e}dP_{Y}\right)}^{\frac{\beta }{\beta -1}}\\ & \geq {\left(\int_{\mathrm{supp}(P_{Y})}{\mathrm{Ren}}_{\alpha }^{\gamma }{\left(X|Y=y\right)}^{\frac{\alpha -1}{\alpha }}dP_{Y}\right)}^{e\frac{\beta }{\beta -1}}\\ & ={\mathrm{Ren}}_{\alpha }^{\gamma }\left(X|Y\right). \end{array}$$

The first inequality above follows from the fact that the unconditional Rényi entropy (probability) decreases (increases) with order. Note that e≥1 when 1<α≤β<∞ and hence the function $r\mapsto r^{e}$ is convex making the second inequality an application of Jensen’s inequality in this case. When 0<α≤β<1, the exponent satisfies 0<e≤1 so the function $r\mapsto r^{e}$ is concave but the outer exponent $\frac{\beta }{\beta -1}$ is negative which turns the (second) inequality in the desired direction. □

### 4.3. Conditioning Reduces Rényi Entropy

As is the case for Shannon entropy, we find that the conditional Rényi entropy obeys monotonicity too; the proof of the theorem below adapts the approach for the discrete case taken in [30] by using Minkowski’s integral inequality.

**Theorem** **2.** *[Monotonicity] Let*α∈[0,∞], *and X be S-valued and*Y,Z*be T-valued random variables. Then,*

$$h_{\alpha }^{\gamma }\left(X|YZ\right)\leq h_{\alpha }^{\gamma }\left(X|Z\right).$$

**Proof.**  We begin by proving the result for an empty *Z*.

First, we deal with the case 1<α<∞. In terms of Rényi probabilities we must show that conditioning increases Rényi probability. Indeed,

(1) $$\begin{array}{cc} {\mathrm{Ren}}_{\alpha }^{\gamma }\left(X|Y\right) & ={\left(\int_{T}{\left[\int_{S}F{\left(x,y\right)}^{\alpha }d\gamma \left(x\right)\right]}^{\frac{1}{\alpha }}d\eta \left(y\right)\right)}^{\frac{\alpha }{\alpha -1}}\\ & \geq {\left[\int_{S}{\left(\int_{T}F\left(x,y\right)d\eta \left(y\right)\right)}^{\alpha }d\gamma \left(x\right)\right]}^{\frac{1}{\alpha }\frac{\alpha }{\alpha -1}}\\ & ={\left(\int_{S}f{\left(x\right)}^{\alpha }d\gamma \left(x\right)\right)}^{\frac{1}{\alpha -1}}={\mathrm{Ren}}_{\alpha }^{\gamma }\left(X\right). \end{array}$$

The inequality above is a direct application of Minkowski’s integral inequality ([44], Theorem 2.4), which generalizes the summation in the standard triangle inequality to integrals against a measure.

For the case 0<α<1, we apply the triangle inequality for 1/α then the fact that now $\frac{1}{\alpha -1}$ is negative flips the inequality in the desired direction:

$$\begin{array}{cc} {\mathrm{Ren}}_{\alpha }^{\gamma }\left(X|Y\right) & ={\left(\int_{T}{\left[\int_{S}F{\left(x,y\right)}^{\alpha }d\gamma \left(x\right)\right]}^{\frac{1}{\alpha }}d\eta \left(y\right)\right)}^{\frac{\alpha }{\alpha -1}}\\ & \geq {\left[\int_{S}{\left(\int_{T}F{\left(x,y\right)}^{\frac{\alpha }{\alpha }}d\eta \left(y\right)\right)}^{\alpha }d\gamma \left(x\right)\right]}^{\frac{1}{\alpha -1}}\\ & ={\left(\int_{S}f{\left(x\right)}^{\alpha }d\gamma \left(x\right)\right)}^{\frac{1}{\alpha -1}}={\mathrm{Ren}}_{\alpha }^{\gamma }\left(X\right). \end{array}$$

Now to extend this to non-empty *Z* we observe the following. Suppose *V* is an *S*-valued random variable, *W* is a *T*-valued random variable and h∈R is such that

$$h_{\alpha }^{\gamma }\left(V|W,Z=z\right)\geq h_{\alpha }^{\gamma }\left(X|Y,Z=z\right)+h,$$

for every *z* in the support of $P_{Z}$. Then

$$h_{\alpha }^{\gamma }\left(V|WZ\right)\geq h_{\alpha }^{\gamma }\left(X|YZ\right)+h.$$

In terms of Renyi probabilities this means that if for every $z\in \mathrm{supp}(P_{Z})$,

$${\mathrm{Ren}}_{\alpha }^{\gamma }\left(V|W,Z=z\right)\leq \frac{{\mathrm{Ren}}_{\alpha }^{\gamma }\left(X|Y,Z=z\right)}{\log h},$$

then

$${\mathrm{Ren}}_{\alpha }^{\gamma }\left(V|WZ\right)\leq \frac{{\mathrm{Ren}}_{\alpha }^{\gamma }\left(X|YZ\right)}{\log h}.$$

Indeed,

$$\begin{array}{cc} {\mathrm{Ren}}_{\alpha }^{\gamma }\left(V|WZ\right) & ={\left(\int_{\mathrm{supp}(Z)}{\mathrm{Ren}}_{\alpha }^{\gamma }{\left(V|W,Z=z\right)}^{\frac{\alpha -1}{\alpha }}dP_{Z}\left(z\right)\right)}^{\frac{\alpha }{\alpha -1}}\\ & \leq {\left(\int_{\mathrm{supp}(Z)}{\left(\frac{{\mathrm{Ren}}_{\alpha }^{\gamma }\left(X|Y,Z=z\right)}{\log h}\right)}^{\frac{\alpha -1}{\alpha }}dP_{Z}\left(z\right)\right)}^{\frac{\alpha }{\alpha -1}}\\ & =\frac{{\mathrm{Ren}}_{\alpha }^{\gamma }\left(X|YZ\right)}{\log h}, \end{array}$$

since the functions $r\mapsto r^{\frac{\alpha -1}{\alpha }}$ and $r\mapsto r^{\frac{\alpha }{\alpha -1}}$ are both strictly increasing or strictly decreasing, based on the value of α. Finally, the claim for non-empty *Z* follows from this observation given we have already demonstrated $h_{\alpha }^{\gamma }\left(X|Y,Z=z\right)\leq h_{\alpha }^{\gamma }\left(X|Z=z\right)$ throughout supp(Z). The cases α=0,∞ are obtained by taking limits. For α=1 this is well-known.□

**Corollary** **1.** *If X→Y→Z forms a Markov chain, then $h_{\alpha }^{\gamma }\left(X|Y\right)\leq h_{\alpha }^{\gamma }\left(X|Z\right)$.*

When we specialize to “empty *Z*” (i.e., the σ-field generated by *Z* being the Borel σ-field on *T*, or not conditioning on anything), we find that “conditioning reduces Rényi entropy”.

**Corollary** **2.** *Let α∈(0,∞). Then $h_{\alpha }^{\gamma }\left(X|Y\right)\leq h_{\alpha }^{\gamma }\left(X\right)$, with equality iff X and Y are independent.*

While the inequality in Corollary 2 follows immediately from Theorem 2, the conditions for equality follow from those for Minkowski’s inequality (which is the key inequality used in the proof of Theorem 2, see, e.g., ([44], Theorem 2.4)): given the finiteness of both sides in the display (1), equality holds if and only if F(x,y)=ϕ(x)ψ(y)γ⊗η-a.e. for some functions ϕ and ψ. In our case, this means that equality holds in $h_{\alpha }^{\gamma }\left(X|Y\right)\leq h_{\alpha }^{\gamma }\left(X\right)$ if and only if *X* and *Y* are independent (α∈(0,1)∪(1,∞)). The corresponding statement for α=1 is well-known.

Since ${\tilde{h}}_{\alpha }^{\gamma }\left(X|Y\right)\leq h_{\alpha }^{\gamma }\left(X|Y\right)$ when 0<α<1, as noted in Section 2, we have “conditioning reduces Rényi entropy” in this case as well.

**Corollary** **3.** ${\tilde{h}}_{\alpha }^{\gamma }\left(X|Y\right)\leq h_{\alpha }^{\gamma }\left(X\right)$, *when*0<α<1.

**Remark** **5.** *The above corollary is not true for large values of α. For a counter-example, see ([28], Theorem 7).*

From the special case when *Y* is discrete random variable taking finitely many values $y_{i}$ with probability $p_{i}$, 1≤i≤n, and the conditional density of *X* given $Y=y_{i}$ is $f_{i}\left(x\right)$, we obtain the concavity of Rényi entropy (for orders below 1) which is already known in the literature.

**Corollary** **4.** *Let*0≤α≤1. *Suppose*$f_{i}$*are densities on S and*$p_{i}$*non-negative numbers,*1≤i≤n*, such that *$\sum_{i}p_{i}=1$*. Then,*$h_{\alpha }^{\gamma }\left(\sum_{i=1}^{n}p_{i}f_{i}\right)\geq \sum_{i=1}^{n}p_{i}h_{\alpha }^{\gamma }\left(f_{i}\right)$.

### 4.4. A chain Rule

In this subsection we deduce a version of the chain rule from Theorem 1. For the discrete case, this has been done by Fehr and Berens in ([30], Theorem 3). If η is a probability measure, then we have $h_{\alpha }^{\gamma }\left(X|Y\right)\geq -D_{\alpha }\left(P_{X,Y}∥\gamma ⊗\eta \right)=h_{\alpha }^{\gamma ⊗\eta }\left(X,Y\right)$. If we relax the condition on η to be a measure under which $P_{Y}$ is absolutely continuous and supported on a set of finite measure, we obtain $h_{\alpha }^{\gamma }\left(X|Y\right)\geq h_{\alpha }^{\gamma ⊗\eta }\left(X,Y\right)-h_{0}^{\eta }\left(Y\right)$. Since this inequality trivially holds true when *Y* is supported on a set of infinite η-measure, we have proved the following inequality that (although weaker being an inequality rather an identity) is reminiscent of the chain rule for Shannon entropy.

**Proposition** **4.** *For any*α>0,

$$h_{\alpha }^{\gamma ⊗\eta }\left(X,Y\right)\leq h_{\alpha }^{\gamma }\left(X|Y\right)+h_{0}^{\eta }\left(Y\right).$$

**Proof.**  Recall that

$${\mathrm{Ren}}_{\alpha }^{\gamma }\left(X|Y\right)={\left[\int_{T}{\left(\int_{S}F{\left(x,y\right)}^{\alpha }d\gamma \left(x\right)\right)}^{\frac{1}{\alpha }}d\eta \left(y\right)\right]}^{\frac{\alpha }{\alpha -1}},$$

where the outer integral can be restricted to the support of $P_{Y}$, which we will keep emphasizing in the first few steps.

$$\begin{array}{cc} {\mathrm{Ren}}_{\alpha }^{\gamma }\left(X|Y\right) & ={\left[\int_{\mathrm{supp}(P_{Y})}{\left(\int_{S}F{\left(x,y\right)}^{\alpha }d\gamma \left(x\right)\right)}^{\frac{1}{\alpha }}d\eta \left(y\right)\right]}^{\frac{\alpha }{\alpha -1}}\\ & ={\left[\int_{\mathrm{supp}(P_{Y})}{\left(\eta \left(\mathrm{supp}\left(P_{Y}\right)\right)\int_{S}F{\left(x,y\right)}^{\alpha }d\gamma \left(x\right)\right)}^{\frac{1}{\alpha }}d\frac{\eta (y)}{\eta (\mathrm{supp}\left(P_{Y}\right))}\right]}^{\frac{\alpha }{\alpha -1}}\\ & ={\left[\int_{\mathrm{supp}(P_{Y})}{\left(\int_{S}{\left(\eta \left(\mathrm{supp}\left(P_{Y}\right)\right)F\left(x,y\right)\right)}^{\alpha }d\gamma \left(x\right)\right)}^{\frac{1}{\alpha }}d\frac{\eta (y)}{\eta (\mathrm{supp}\left(P_{Y}\right))}\right]}^{\frac{\alpha }{\alpha -1}}. \end{array}$$

By Jensen’s inequality, when α>1,

$$\begin{array}{cc} {\mathrm{Ren}}_{\alpha }^{\gamma }\left(X|Y\right) & \leq {\left[\int_{\mathrm{supp}(P_{Y})}\left(\int_{S}{\left(\eta \left(\mathrm{supp}\left(P_{Y}\right)\right)F\left(x,y\right)\right)}^{\alpha }d\gamma \left(x\right)\right)d\frac{\eta (y)}{\eta (\mathrm{supp}\left(P_{Y}\right))}\right]}^{\frac{1}{\alpha -1}}\\ & =\eta \left(\mathrm{supp}\left(P_{Y}\right)\right){\left[\int_{\mathrm{supp}(P_{Y})}\int_{S}F{\left(x,y\right)}^{\alpha }d\gamma \left(x\right)d\eta \left(y\right)\right]}^{\frac{1}{\alpha -1}}\\ & =\eta \left(\mathrm{supp}\left(P_{Y}\right)\right){\mathrm{Ren}}_{\alpha }^{\gamma ⊗\eta }\left(X,Y\right). \end{array}$$

Note that the above calculation also holds when α∈(0,1) because even though Jensen’s inequality is flipped because $\frac{1}{\alpha }$ is now convex, the inequality flips again, now to the desired side, since the exponent $\frac{\alpha }{\alpha -1}$ is negative. Taking logarithms and hitting both sides with a minus sign concludes the proof.□

**Remark** **6.** *These inequalities are tight. Equality is attained when X, Y are independent and Y is uniformly distributed on finite support.*

### 4.5. Sensitivity to Reference Measure

**Proposition** **5.** *Suppose* $\frac{d\gamma }{d\mu }=\psi$ *which is bounded by a number M. Then* $h_{\alpha }^{\mu }\left(X|Y\right)\geq h_{\alpha }^{\gamma }\left(X|Y\right)-\log M.$

**Proof.**  Then the joint density of (X,Y) under the measure μ⊗μ becomes F(x,y)ψ(x)ψ(y) if the joint density was F(x,y) under γ. Now suppose α≥1 then,

$$\begin{array}{c} h_{\alpha }^{\mu }\left(X|Y\right)\\ \;=\frac{-\alpha }{\alpha -1}\log\left[\int_{T}{\left(\int_{S}F{\left(x,y\right)}^{\alpha }\psi {\left(x\right)}^{\alpha }\psi {\left(y\right)}^{\alpha }d\mu \left(x\right)\right)}^{\frac{1}{\alpha }}d\mu \left(y\right)\right]\\ \;=\frac{-\alpha }{\alpha -1}\log\left[\int_{T}{\left(\int_{S}F{\left(x,y\right)}^{\alpha }\psi {\left(x\right)}^{\alpha }d\mu \left(x\right)\right)}^{\frac{1}{\alpha }}\psi \left(y\right)d\mu \left(y\right)\right]\\ \;=\frac{-\alpha }{\alpha -1}\log\left[\int_{T}{\left(\int_{S}F{\left(x,y\right)}^{\alpha }\psi {\left(x\right)}^{\alpha -1}d\gamma \left(x\right)\right)}^{\frac{1}{\alpha }}d\gamma \left(y\right)\right]\\ \;\geq \frac{-\alpha }{\alpha -1}\left(\log\left[\int_{T}{\left(\int_{S}F{\left(x,y\right)}^{\alpha }d\gamma \left(x\right)\right)}^{\frac{1}{\alpha }}d\gamma \left(y\right)\right]+\log M^{\frac{\alpha -1}{\alpha }}\right)\\ \;=h_{\alpha }^{\gamma }\left(X|Y\right)-\log M. \end{array}$$

If α∈(0,1), the same inequality holds if μ is also absolutely continuous w.r.t. γ.□

## 5. Notions of α-Mutual Information

Arimoto [39] used his conditional Rényi entropy to define a mutual information that we extend to the general setting as follows.

**Definition** **5.** *Let X be an S-valued random variable and let Y be a T-valued random variable, with a given joint distribution. Then, we define*

$$I_{\alpha }^{\left(\gamma \right)}\left(X⇝Y\right)=h_{\alpha }^{\gamma }\left(X\right)-h_{\alpha }^{\gamma }\left(X|Y\right).$$

We use the squiggly arrow to emphasize the lack of symmetry in *X* and *Y*, but nonetheless to distinguish from the notation for directed mutual information, which is usually written with a straight arrow. By Corollary 2, for α∈(0,∞), $I_{\alpha }^{\left(\gamma \right)}\left(X⇝Y\right)=0$ if and only if *X* and *Y* are independent. Therefore $I_{\alpha }^{\left(\gamma \right)}\left(X⇝Y\right)$, for any choice of reference measure γ, can be seen as a measure of dependence between *X* and *Y*.

Let us discuss a little further the validity of $I_{\alpha }^{\left(\gamma \right)}\left(X⇝Y\right)$ as a dependence measure. If the conditional distributions are denoted by $P_{X|Y=y}$ as in Remark 1, using the fact that $h_{\alpha }^{\nu }\left(Z\right)=-D_{\alpha }\left(Z∥\nu \right)$ for any random variable *Z*, we have for any α∈(0,1)∪(1,∞) that

$$\begin{array}{cc} I_{\alpha }^{\left(\gamma \right)}\left(X⇝Y\right) & =h_{\alpha }^{\gamma }\left(X\right)-h_{\alpha }^{\gamma }\left(X|Y\right)\\ & =\frac{\alpha }{1-\alpha }\log e^{-\frac{1-\alpha }{\alpha }D_{\alpha }\left(P_{X}∥\gamma \right)}-\frac{\alpha }{1-\alpha }\log\int_{T}e^{-\frac{1-\alpha }{\alpha }D_{\alpha }\left(P_{X|Y=y}∥\gamma \right)}dP_{Y}\left(y\right)\\ & =-\frac{\alpha }{1-\alpha }\log\int_{T}e^{-\frac{1-\alpha }{\alpha }\left[D_{\alpha }\left(P_{X|Y=y}∥\gamma \right)-D_{\alpha }\left(P_{X}∥\gamma \right)\right]}dP_{Y}\left(y\right). \end{array}$$

Furthermore, when α∈(0,1), by ([29], Proposition 2), we may also write

$$\begin{array}{cc} I_{\alpha }^{\left(\gamma \right)}\left(X⇝Y\right) & =-\frac{\alpha }{1-\alpha }\log\int_{T}e^{-[D_{1-\alpha }\left(\gamma ∥P_{X|Y=y}\right)-D_{1-\alpha }\left(\gamma ∥P_{X}\right)]}dP_{Y}\left(y\right). \end{array}$$

Note that Rényi divergence is convex in the second argument (see [29], Theorem 12) when α∈(0,1), and the last equation suggests that Arimoto’s mutual information can be seen as a quantification of this convexity gap.

**Figure 1:**
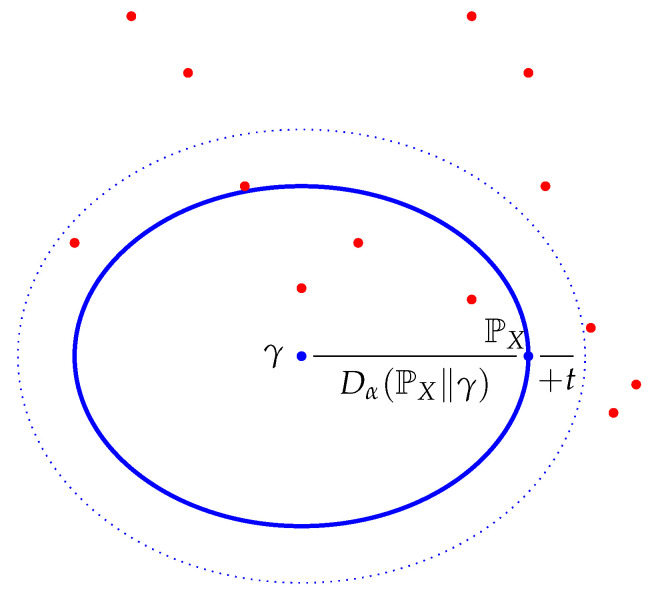
A schematic diagram showing how large $I_{\alpha }^{\left(\gamma \right)}\left(X⇝Y\right)$, for a fixed α<1, demonstrates strong dependence between *X* and *Y*: the space depicted is the space of probability measures on *S*, including γ, $P_{X}$, and the red dots representing the conditional distributions of *X* given that *Y* takes different values in *T*. The $D_{\alpha }$-balls around γ are represented by ellipses to emphasize that the geometry is non-Euclidean and in fact, non-metric. When $I_{\alpha }^{\left(\gamma \right)}\left(X⇝Y\right)$ is large, there is a significant probability that *Y* takes values such that the corresponding conditional distributions of *X* lie outside the larger $D_{\alpha }$-ball, and therefore far from the (unconditional) distribution $P_{X}$ of *X*.

One can also see clearly from the above expressions why this quantity controls, at least for α∈(0,1), the dependence between *X* and *Y*: indeed, one has for any α∈(0,1) and any t>0 that,

$$\begin{array}{cc} P_{Y}\left\{D_{\alpha }\left(P_{X|Y}∥\gamma \right)-D_{\alpha }\left(P_{X}∥\gamma \right)<t\right\} & =P_{Y}\left\{e^{-\beta [D_{\alpha }\left(P_{X|Y}∥\gamma \right)-D_{\alpha }\left(P_{X}∥\gamma \right)]}>e^{-\beta t}\right\}\leq e^{\beta t}e^{-\beta I_{\alpha }^{\left(\gamma \right)}\left(X⇝Y\right)}, \end{array}$$

where the inequality comes from Markov’s inequality, and we use $\beta =\frac{1-\alpha }{\alpha }$. Thus, when $I_{\alpha }^{\left(\gamma \right)}\left(X⇝Y\right)$ is large, the probability that the conditional distributions of *X* given *Y* cluster at around the same “Rényi divergence” distance from the reference measure γ as the unconditional distribution of *X* (which is of course a mixture of the conditional distributions) is small, suggesting a significant “spread” of the conditional distributions and therefore strong dependence. This is illustrated in Figure 1. Thus, despite the dependence of $I_{\alpha }^{\left(\gamma \right)}\left(X⇝Y\right)$ on the reference measure γ, it does guarantee strong dependence when it is large (at least for α<1). When $\alpha \to 1^{-}$ we have β→0, and consequently the upper bound $e^{\beta t}e^{-\beta I_{\alpha }^{\left(\gamma \right)}\left(X⇝Y\right)}\to 1$ making the inequality trivial.

The “mutual information” quantity $I_{\alpha }^{\left(\gamma \right)}\left(X⇝Y\right)$ clearly depends on the choice of the reference measure γ. Nonetheless, there are 3 families of Rényi mutual informations that are independent of the choice of reference measure, which we now introduce.

**Definition** **6.** *Fix*α≥0.

*1*. *The Lapidoth-Pfister α-mutual information is defined as*
  $$J_{\alpha }\left(X;Y\right):=\min_{\mu \in P(S),\nu \in P(T)}D_{\alpha }\left(P_{X,Y}∥\mu ⊗\nu \right).$$
*2*. *The Augustin-Csiszár α-mutual information is defined as*
  $$K_{\alpha }^{X⇝Y}\left(P_{X,Y}\right)=\min_{\mu \in P(T)}E_{X}D_{\alpha }\left(P_{Y|X}\left(\cdot |X\right)∥\mu \right).$$
*3*. *Sibson’s α-mutual information is defined as*
  $$I_{\alpha }^{X⇝Y}\left(P_{X,Y}\right)=\min_{\mu \in P(T)}D_{\alpha }\left(P_{X,Y}∥P_{X}⊗\mu \right).$$

The quantity $J_{\alpha }$ was recently introduced by Lapidoth and Pfister as a measure of independence in [45] (cf., [25,27,32]). The Augustin-Csiszár mutual information was originally introduced in [40] by Udo Augustin with a slightly different parametrization, and gained much popularity following Csiszár’s work in [21]. For a discussion on early work on this quantity and applications also see [42] and references therein. Both [40] and [42] treat abstract alphabets however the former is limited to α∈(0,1) while the latter treats all α∈(0,∞). Sibson’s definition originates in [38] where he introduces $I_{\alpha }$ in the form of *information radius* (see, e.g, [33]), which is often written in terms of Gallager’s function (from [46]). Since all the quantities in the above definition are stated in terms of Rényi divergences not involving the reference measure γ, they themselves are independent of the reference measure. Their relationship with the Rényi divergence also shows that all of them are non-negative. Moreover, putting $\mu =P_{X},\nu =P_{Y}$ in the expression for $J_{\alpha }$ and $\mu =P_{Y}$ in expressions for $K_{\alpha }$ and $I_{\alpha }$ when X,Y are independent shows that they all vanish under independence.

While these notions of mutual information are certainly not equal to $I_{\alpha }^{\left(\gamma \right)}$ in general when α≠1, they do have a direct relationship with conditional Rényi entropies, by varying the reference measure.

Since $\min_{\mu }\min_{\nu }D_{\alpha }\left(P_{X,Y}∥\mu ⊗\nu \right)=\min_{\mu }-h_{\alpha }^{\mu }\left(X|Y\right)=-\max_{\mu }h_{\alpha }^{\mu }\left(X|Y\right)$, where all optimizations are done over probability measures, we can write Lapidoth and Pfister’s mutual information as

$$J_{\alpha }\left(X;Y\right)=-\max_{\mu }h_{\alpha }^{\mu }\left(X|Y\right)=\min_{\mu }-\frac{\alpha }{1-\alpha }\log\int_{T}e^{-\frac{1-\alpha }{\alpha }D_{\alpha }\left(P_{X|Y=y}∥\mu \right)}dP_{Y}\left(y\right).$$

Note that it is symmetric by definition: $J_{\alpha }\left(X;Y\right)=J_{\alpha }\left(Y;X\right)$, which is why we do not use squiggly arrows to denote it. By writing down Rényi divergence as Rényi entropy w.r.t. reference measure, Augustin-Csiszár’s $K_{\alpha }$ can be recast in a similar form, this time using the average Rényi entropy of the conditionals instead of Arimoto’s conditional Rényi entropy,

$$K_{\alpha }^{X⇝Y}=-\max_{\mu }E_{X}h_{\alpha }^{\mu }\left(Y|X=x\right)=-\max_{\mu }{\tilde{h}}_{\alpha }^{\mu }\left(Y|X\right).$$

In light of Theorem 1, Sibson’s mutual information can clearly be written in terms of conditional Rényi entropy as

$$I_{\alpha }^{X⇝Y}=-h_{\alpha }^{P_{X}}\left(X|Y\right).$$

This leads to the observation that Sibson’s mutual information can be seen as a special case of Arimoto’s mutual information, when the reference measure is taken to be the distribution of *X*:

$$\begin{array}{c} I_{\alpha }^{X⇝Y}=-h_{\alpha }^{P_{X}}\left(X|Y\right)\\ =h_{\alpha }^{P_{X}}\left(X\right)-h_{\alpha }^{P_{X}}\left(X|Y\right)=I_{\alpha }^{\left(P_{X}\right)}\left(X⇝Y\right). \end{array}$$

For the sake of comparision with the corresponding expression for $I_{\alpha }^{\left(\gamma \right)}\left(X⇝Y\right)$, we also write $I_{\alpha }^{X⇝Y}$ as

$$I_{\alpha }^{X⇝Y}=-\frac{\alpha }{1-\alpha }\log\int_{T}e^{-\frac{1-\alpha }{\alpha }D_{\alpha }\left(P_{X|Y=y}∥P_{X}\right)}dP_{Y}\left(y\right).$$

The following inequality, which relates the three families when α≥1, turns out to be quite fruitful.

**Theorem** **3.** *For* α≥1 *, we have*

$$K_{\alpha }^{X⇝Y}\leq J_{\alpha }\left(X;Y\right)\leq I_{\alpha }^{X⇝Y}.$$

**Proof.**  Suppose α≥1. Then, $h_{\alpha }^{\mu }\left(Y|X\right)\leq {\tilde{h}}_{\alpha }^{\mu }\left(Y|X\right)$ so that

$$\begin{array}{cc} K_{\alpha }^{X⇝Y}=-\max_{\mu }{\tilde{h}}_{\alpha }^{\mu }\left(Y|X\right) & \leq -\max_{\mu }h_{\alpha }^{\mu }\left(Y|X\right)\\ & =J_{\alpha }\left(Y;X\right)=J_{\alpha }\left(X;Y\right). \end{array}$$

Moreover,

$$J_{\alpha }\left(X;Y\right)=-\max_{\mu }h_{\alpha }^{\mu }\left(X|Y\right)\leq -h_{\alpha }^{P_{X}}\left(X|Y\right)=I_{\alpha }^{X⇝Y},$$

which completes the proof. □

**Remark** **7.** *When*α∈(0,1)*, from the straightforward observation*$J_{\alpha }\left(X;Y\right)\leq I_{\alpha }^{X⇝Y}$*and [21], we have*$J_{\alpha }\left(X;Y\right)\leq I_{\alpha }^{X⇝Y}\leq K_{\alpha }^{X⇝Y}$.

We note that the relation between $K_{\alpha }^{X⇝Y}$ and $I_{\alpha }^{X⇝Y}$ (in the finite alphabet case) goes back to Csiszár [21]. In the next and final section, we explore the implications of Theorem 3 for various notions of capacity.

## 6. Channels and Capacities

We begin by defining channel and capacities. Throughout this section, assume α≥1.

**Definition** **7.** *Let* (A,A),(B,B) *be measurable spaces. A function* W:A×B→R *is called a probability kernel or a channel from the input space* (A,A) *to the output space* (B,B) *if*

*1*. *For all* a∈A *, the function* W(·|a):B→R *is a probability measure on* (B,B) *, and*
*2*. *For every* V∈B *, the function* W(V|·):A→R *is a* A *-measurable function.*

In our setting, the conditional distributions *X* given Y=y define a channel *W* from $\mathrm{supp}(P_{Y})$ to *S*. In terms of this, one can write a density-free expression for the conditional Rényi entropy:

$$h_{\alpha }^{\gamma }\left(X|Y\right)=\frac{\alpha }{1-\alpha }\log\int_{T}e^{-\frac{1-\alpha }{\alpha }D_{\alpha }\left(W\left(\cdot |y\right)∥\gamma \right)}dP_{Y}\left(y\right).$$

**Definition** **8.** *Let* (B,B) *be a measurable space and* W⊆P(B) *a set of probability measures on B. Following [36], define the order-α Rényi radius of* W *relative to* q∈P(B) *by*

$$S_{\alpha ,W}\left(q\right)=\underset{w\in W}{\sup}D_{\alpha }\left(w∥q\right).$$

*The order-α Rényi radius of* W *is defined as*

$$S_{\alpha }\left(W\right)=\underset{q\in P(B)}{\inf}S_{\alpha ,W}\left(q\right).$$

Given a joint distribution $P_{X,Y}$ of (X,Y), one can consider the quantities $I_{\alpha }^{X⇝Y},I_{\alpha }^{Y⇝X},K_{\alpha }^{X⇝Y},K_{\alpha }^{Y⇝X}$ and $J_{\alpha }\left(X;Y\right)$, as functions of an “input” distribution $P_{X}$ and the channel from *X* to *Y* (i.e., the probability kernel formed by the conditional distributions of *Y* given X=x). For example, one can consider $I_{\alpha }^{Y⇝X}=\inf_{\nu \in P(S)}D_{\alpha }\left(P_{X,Y}∥\nu ⊗P_{Y}\right)$ as a function of $P_{X}$ and the probability kernel formed by the conditional distributions of *Y* given X=x. Under this interpretation, we first define various capacities.

**Definition** **9.** *Given a channel W from X to Y, we define capacities of order α by*

*1*. $C_{K,\alpha }^{X⇝Y}\left(W\right)=\sup_{P\in P(S)}K_{\alpha }^{X⇝Y}\left(P,W\right)$,
*2*. $C_{J,\alpha }\left(W\right)=\sup_{P\in P(S)}J_{\alpha }\left(P,W\right)$ *, and*
*3*. $C_{I,\alpha }^{X⇝Y}\left(W\right)=\sup_{P\in P(S)}I_{\alpha }^{X⇝Y}\left(P,W\right)$.

Theorem 3 allows us to extend ([31], Theorem 5) to include the capacity based on the Lapidoth-Pfister mutual information.

**Theorem** **4.** *Let* α≥1 *, and fix a channel W from X to Y. Then,*

$$\begin{array}{cc} C_{K,\alpha }^{Y⇝X}\left(W\right)\leq C_{I,\alpha }^{X⇝Y}\left(W\right) & =C_{J,\alpha }\left(W\right)=C_{K,\alpha }^{X⇝Y}\left(W\right)=S_{\alpha }\left(W\right)\leq C_{I,\alpha }^{Y⇝X}\left(W\right). \end{array}$$

**Proof.**  Theorem 3 implies that,

$$\underset{P\in P(S)}{\sup}K_{\alpha }^{X⇝Y}\left(P,W\right)\leq \underset{P\in P(S)}{\sup}J_{\alpha }\left(P,W\right)\leq \underset{P\in P(S)}{\sup}I_{\alpha }^{X⇝Y}\left(P,W\right),$$

that is,

$$C_{K,\alpha }^{X⇝Y}\left(W\right)\leq C_{J,\alpha }\left(W\right)\leq C_{I,\alpha }^{X⇝Y}\left(W\right).$$

It was shown by Csiszár [21] in the finite alphabet setting (in fact, he showed this for all α>0) that $C_{I,\alpha }^{X⇝Y}\left(W\right)=C_{K,\alpha }^{X⇝Y}\left(W\right)$. Nakiboğlu demonstrates $C_{I,\alpha }^{X⇝Y}\left(W\right)=S_{\alpha }\left(W\right)$ in [36] and $C_{K,\alpha }^{X⇝Y}\left(W\right)=S_{\alpha }\left(W\right)$ in [42] for abstract alphabets. Putting all this together, we have

$$C_{I,\alpha }^{X⇝Y}\left(W\right)=C_{J,\alpha }\left(W\right)=C_{K,\alpha }^{X⇝Y}\left(W\right)=S_{\alpha }\left(W\right).$$

Finally, using the symmetry of $J_{\alpha }$ and Theorem 3 in a similar fashion again, we get

$$C_{K,\alpha }^{Y⇝X}\left(W\right)\leq C_{J,\alpha }\left(W\right)\leq C_{I,\alpha }^{Y⇝X}\left(W\right).$$

This completes the proof.□

The two inequalities in the last theorem cannot be improved to be equalities, this follows from a counter-example communicated to the authors by C. Pfister. Note that this theorem corrects Theorem V.1 in [41].

Since $J_{\alpha }\left(X;Y\right)$ is no longer sandwiched between $K_{\alpha }^{X⇝Y}$ and $I_{\alpha }^{X⇝Y}$ when α∈(0,1) the same argument cannot be used to deduce the equality of various capacities in this case. However, when α∈[1/2,1), a direct demonstration proves that the Lapidoth-Pfister capacity of a channel equals Rényi radius when the state spaces are finite.

**Theorem** **5.** *Let* $\alpha \in [\frac{1}{2},1)$ *, and fix a channel W from X to Y where X and Y take values in finite sets S and T respectively. Then,*

$$\underset{P\in P(S)}{\sup}J_{\alpha }\left(P,W\right)=S_{\alpha }\left(W\right).$$

**Proof.**  We continue using integral notation instead of summation. Note that,

$$J_{\alpha }\left(X;Y\right)=-\max_{\mu \in P(T)}-\beta \log\int_{S}e^{\frac{1}{\beta }D_{\alpha }\left(W\left(x\right)∥\mu \right)}dP\left(x\right)=\min_{\mu \in P(T)}\beta \log\int_{S}e^{\frac{1}{\beta }D_{\alpha }\left(W\left(x\right)∥\mu \right)}dP\left(x\right),$$

where $\beta =\frac{\alpha }{\alpha -1}.$ We consider the function $f\left(P,\mu \right)=\beta \log\int_{S}e^{\frac{1}{\beta }D_{\alpha }\left(W\left(x\right)∥\mu \right)}dP\left(x\right)$ defined on P(S)×P(T). Observe that the function $g\left(P,\mu \right)=-e^{\frac{1}{\beta }f\left(P,\mu \right)}=-\int_{S}e^{\frac{1}{\beta }D_{\alpha }\left(W\left(x\right)∥\mu \right)}dP\left(x\right)$ has the same minimax properties as *f*. We make the following observations about this function.

- g is linear in P.
- *g is convex in μ*.
  Follows from the proof in ([27], Lemma 17).
- *g is continuous in each of the variables P and μ.* Continuity in μ follows from continuity of $D_{\alpha }$ in the second coordinate (see, for example, in [29]) whereas continuity in *P* is a consequence of linearity of the integral (summation).

The above observations ensure that we can apply von Neumann’s convex minimax theorem to *g*, and therefore to *f* to conclude that

$$\underset{P}{\sup}J_{\alpha }\left(P,W\right)=\underset{P}{\sup}\min_{\mu }f\left(P,\mu \right)=\min_{\mu }\underset{P}{\sup}f\left(P,\mu \right).$$

For a fixed μ however, $\sup_{P}f\left(P,\mu \right)=\sup_{P}\beta \log\int e^{\frac{1}{\beta }D_{\alpha }\left(W\left(x\right)∥\mu \right)}dP\left(x\right)=\sup_{x}D_{\alpha }\left(W\left(x\right)∥\mu \right)$ (the RHS is clearly bigger than the LHS, for the other direction use measures $P=\delta_{x_{n}}$ where $x_{n}$ is a supremum achieving sequence for the RHS). This shows that when α≥1/2 the capacity coming from the $J_{\alpha }\left(X;Y\right)$ equals the Rényi radius if the state spaces are finite.□

Though we do not treat capacities coming from Arimoto’s mutual information in this paper due to its dependence on a reference measure, a remark can be made in this regard following B. Nakiboğlu’s [43] observation that Arimoto’s mutual information w.r.t. γ of a joint distribution (X,Y) can be written as a Sibson mutual information of some input probability measure *P* and the channel *W* from *X* to *Y* corresponding to $P_{X,Y}$. Let X,Y denote the marginals of (X,Y). As before there are reference measures γ on the state space *S* of *X*. Let *P* denote the probability measure on *S* with density $\frac{dP}{d\gamma }=e^{\left(1-\alpha \right)D_{\alpha }\left(P_{X}∥\gamma \right)}{\left(\frac{dP_{X}}{d\gamma }\right)}^{\alpha }$. Then a calculation shows that

$$I_{\alpha }^{\left(\gamma \right)}\left(X⇝Y\right)=I_{\alpha }^{X⇝Y}\left(P,W\right).$$

Therefore, it follows that if a reference measure γ is fixed, then the capacity of order α of a channel *W* calculated from Arimoto’s mutual information will be less than the capacity based on Sibson mutual information (which equals the Rényi radius of *W*).
